# Strategies to Evaluate Synchronous Carcinomas of the Colon and Rectum in Patients That Present for Emergent Surgery

**DOI:** 10.1155/2013/309439

**Published:** 2013-02-06

**Authors:** Jennifer L. Agnew, Benjamin Abbadessa, I. Michael Leitman

**Affiliations:** Department of Surgery, Albert Einstein College of Medicine, Beth Israel Medical Center, 10 Union Square East, Suite 2M, New York, NY 10003, USA

## Abstract

It is not always possible to evaluate patients that present acutely with carcinoma of the colon and rectum for synchronous lesions. Patients that require emergent surgery necessitate urgent and efficient operation. Patients with lower gastrointestinal bleeding, perforation, or obstruction represent a challenging subset of patients with colorectal cancer. An organized approach to these patients in the effort not to overlook a synchronous carcinoma is important. The present paper provides an evidenced-based approach to this special situation.

## 1. Background

Colorectal cancer is the third most common malignancy worldwide and the fourth most common in the United States, with estimated 146,970 new cases diagnosed in 2009. In the United States, approximately 49,920 cancer-related deaths were attributed to a colorectal malignancy in 2009, making it second only to mortality from lung cancer [1]. Despite the high incidence of colorectal carcinoma that is diagnosed yearly, the majority of lesions are resected with curative intent (70%–80%) and colorectal cancer-related deaths account for 20%–30% of those diagnosed and treated surgically, making it a highly curable malignancy if identified in its early-stages. Approximately, 55% of colorectal cancers diagnosed on screening are found to present with Stage I or II disease [2, 3]. Patients with Stage I or Stage II colon cancer have a greater than 60% 5-year survival, and for patients with Stage I or Stage II rectal cancer, there is a greater than 50% 5-year survival [4, 5]. Because of this, colorectal screening exams in asymptomatic patients have become the recommended standard of care for average risk patients at the age of 50 and at the age of 40 or younger for those in the moderate and high risk groups. 

Approximately, 70% of colorectal lesions occur distal to the splenic flexure, and looking at colon cancer alone, approximately 25% are found in the sigmoid, 10% at the rectosigmoid junction, and 4%–6% located in the descending colon [6]. Anatomically, the left colon has a smaller diameter than the right, and as a result, left-sided carcinomas can cause varying degrees of intraluminal occlusion and patients more frequently present with obstructive symptoms. Large bowel obstructions present a challenging clinical scenario for the physician in the diagnosis, operative management, and the timing of colonic surveillance. Patients presenting with advanced lesions causing partial or high-grade large bowel obstruction commonly have a distant history or no history of previous colonic surveillance. This fact is of clinical importance because the diagnosis of a primary colorectal malignancy is accompanied by an overall incidence of synchronous colorectal cancers between 2% and 10% and a frequency of synchronous adenomatous polyps ranging from 15% to 50% [7–13]. Interestingly, when compared by stage, no statistically significant difference has been found in the overall and disease-free survival between synchronous and primary cancers [14, 15]. 

**Figure 1:**
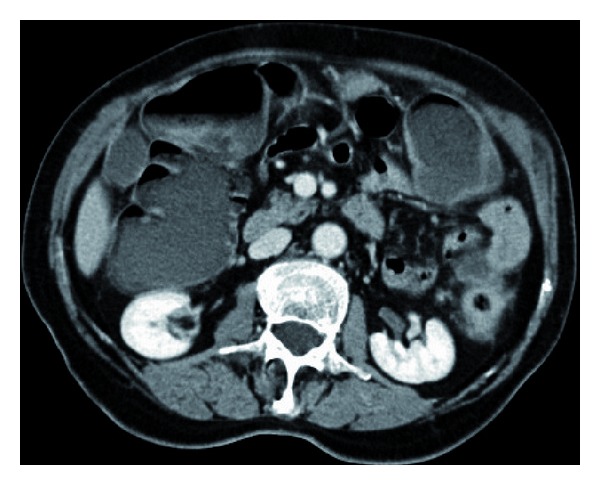
CT scan of abdomen in a patient with obstructing carcinoma of the sigmoid colon.

**Figure 2:**
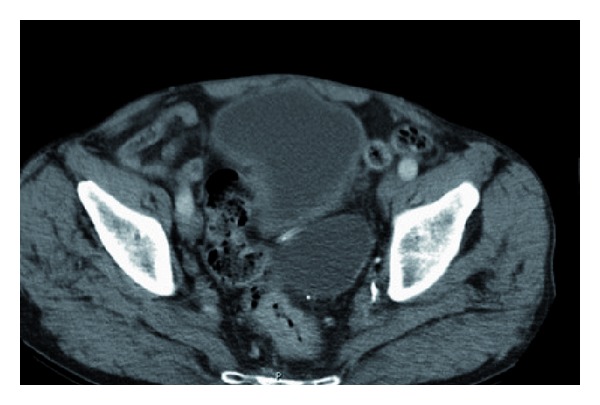
CT scan of a patient with obstructing carcinoma of the rectum.

High-grade or completely obstructing lesions may require emergent surgery, and preoperative colonoscopic surveillance by traditional colonoscopy, CT colonography, or barium enema is typically not recommended due to the lack of accuracy. Colonoscopy has a high rate of unsuccessful passage of the colonoscope as well as the risk of colonoscopic perforation at the site of obstruction or the more proximal cecum secondary to barotrauma. This particular scenario leaves the patient without adequate visualization and evaluation of the proximal colon prior to their operative resection (Figures 1 and 2). The issue of when and how to best monitor these patients is still debated and evolving, with the recent literature advocating intraoperative, on-table colonic lavage and colonoscopy. The current literature and management options for this unique clinical scenario are presented (Table 1). 

## 2. Preoperative

Colonoscopic screening and surveillance has been proven to be a diagnostic and therapeutic modality that can result in an early diagnosis of colorectal cancer at a curable stage as well as identify and remove premalignant polypoid lesions. Quality control measures require cecal intubation during colonoscopy to classify the study as satisfactory and complete, thus decreasing the likelihood of failure to detect malignant or premalignant lesions. An incomplete colonoscopy is defined as failure of cecal intubation (or ileocolonic anastomosis) with nonvisualization of anatomic features (when present), such as the ileocecal valve, appendiceal orifice, or terminal ileum. Using a population-based cohort, Neerincx and colleagues investigated the rates and reasons for incomplete colonoscopy, as well as the subsequent work-up conducted to evaluate the nonvisualized colon, and the incidence of premalignant and cancerous lesions missed on incomplete colonoscopy. Incomplete colonoscopy was reported in 9.7% of patients. Several factors were cited to explain the occurrence, most commonly looping scope/excessively long colon (dolichocolon) (20.4%), patient discomfort (15.3%), obstructing tumor (13.9%), and insufficient bowel preparation (12.1%), with stenosis (3.7%) and severe inflammation (3.5%) being less common. During an 18-month follow-up period after incomplete index colonoscopy, secondary examinations were performed in 54.4% of these patients. Patients with incomplete colonoscopies due to stenosis (78.9%), severe inflammation (77.8%), and obstructing tumor (74.6%) were most likely to receive a secondary examination during the follow-up period. Barium enema was found to be the most common secondary investigation (47.5%) [16], despite its low diagnostic yield and miss rate for colorectal cancer detection, which has been reported as high as 22% [17]. Other modalities for secondary colon examination included repeat colonoscopy (20.5%), computed tomography colonography (4.3%), abdominal computed tomography (20.9%), and surgical exploration (6.8%). In individuals who underwent follow-up colonic evaluation after incomplete endoscopy, colorectal cancer was diagnosed in 3.5% of patients, with advanced adenoma found in 0.8% of patients, therefore noting overall that advanced neoplasia went undetected in 4.3% of patients due to incomplete colonoscopy [16]. 

As described by Neerincx et al., obstructing colorectal tumors may lead to incomplete colonoscopy due to inability to pass the colonoscope beyond the lesion. Morrin and colleagues advocated for the use of virtual computed tomographic (CT) colonography to image and detect synchronous lesions in the colon proximal to an obstructing colorectal lesion. Developed in the 1990s, virtual CT colonography uses ionizing radiation to create endoluminal radiologic images in multiple planes of an air-insufflated, cleansed colon, while simultaneously imaging extracolonic regions of the body. Using this modality, lesions greater than 1 cm can be detected with similar accuracy to conventional colonoscopy and with detection rates superior to barium enema. In the Morrin et al. study, virtual CT colonography correctly staged 81% of colorectal cancers. In patients with incomplete colonoscopy, 97%–100% of all colonic segments were adequately visualized on virtual CT colonography, serving as an accurate preoperative imaging option that could be used to investigate for synchronous proximal lesions, thus assisting with operative planning [18–20]. In a study reported by Wong et al., air-inflated magnetic resonance colonoscopy (MRC) was as accurate for identification of colonic lesions as colonoscopy [21]. As described earlier, while colonoscopy, barium enema, and CT colonography are widely accepted as the principle screening tools for the detection of colorectal cancer, they may be contraindicated and therefore not frequently performed during the hospitalization of patients in need of emergent resection for large bowel obstruction due to carcinoma. 

In the acute setting of complete or near-total colonic obstruction, where many patients will require an emergent operation, preoperative evaluation of the colon may not be possible. Palpation of the colon and intraoperative colonic lavage and on-table colonoscopy are the only current diagnostic and therapeutic modalities available in the immediate perioperative time period. Although preoperative CT imaging may identify a proximal synchronous lesion(s) along with a high-grade, distal symptomatic obstruction, therapeutic colonoscopic intervention would be delayed until months postoperatively unless intraoperative techniques are used. 

## 3. Intraoperative

Throughout history, intent-to-cure surgical resection has remained the treatment of choice for localized regional colorectal cancer. Surgical resection of obstructing carcinomas of the colon is associated with significant morbidity and mortality [22]. However, surgical management of completely obstructive left-sided colon cancer remains a topic of discussion, particularly as operative techniques and technological advancements have evolved over time. Of major concern is the theory that failure to detect a synchronous lesion proximal to the site of obstruction may lead to inadequate operative procedures at the time of initial surgical intervention, thus leaving unaddressed pathology behind and necessitating subsequent therapeutic interventions, including possible repeat surgical resection.

Historically, early studies favored performing a subtotal colectomy in the setting of left colon obstruction secondary to tumor [23, 24]. At that time, subtotal colectomy allowed removal of synchronous cancers and prevented possible development of metachronous lesions in the colon proximal to an obstructing lesion that had not been properly evaluated endoscopically in the preoperative period. However, depending on the length of remaining colon and rectum, frequent bowel movements and even fecal incontinence have been reported in patients who underwent subtotal colectomy [25].

Over time, the concept of a one-stage resection with intraoperative colonic lavage and primary anastomosis evolved as a potential surgical alternative to subtotal colectomy. On-table colonic irrigation was first described by Muir in 1968. Before that time, when a segmental resection was performed on an unprepared colon, standard practice was to create a colostomy (Hartmann's procedure) at the initial laparotomy. In the uncleansed colon, primary anastomosis was deferred at the time of initial surgery due to the anticipated risk of forceful stress associated with passing a solid load across a newly created surgical anastomosis. Acknowledging the known advantages of operating on a prepped colon, Muir investigated feasible techniques to allow a primary anastomosis to be performed when operating on a colon with a high fecal load preoperatively. Since proper preoperative preparation of the bowel can be difficult or impossible to achieve when an obstructing colorectal lesion is present, Muir proposed washing out the obstructed colon at the time of operation. The standard dissection and mobilization are performed as usual, the bowel is clamped and divided distal to the lesion, and the mobilized intestinal segment containing the lesion was inserted into a sterile polythene bag. Proximal to the lesion, a tube was inserted, and irrigation solution was instilled to lavage the colon. The feculent irrigation waste was subsequently drained out through the same tubing. The irrigation tubing was then removed, the lesion resected, and the operation completed with a primary anastomosis on the clean and empty colon [26].

Modifying Muir's lavage technique, Dudley et al. proposed another method of on-table colonic irrigation in 1980. The region of the lesion was dissected, and the bowel was divided distal to the lesion. The proximal bowel was mobilized and a sterile, rigid plastic tube was inserted through the cut end of this portion of bowel. A small enterotomy in the terminal ileum was made, through which a Foley catheter was inserted and passed across the ileocecal valve. The balloon was inflated, and the catheter was secured into place with a purse-string suture. Irrigation solution was instilled antegrade through the Foley catheter, and feculent waste was drained through the rigid plastic tube distally. After the colon was determined to be clean, the specimen was resected, and primary anastomosis of the bowel was completed. The Foley catheter was either removed and the enterotomy closed after resection of the lesion, or the catheter was brought out through a stab wound in the skin to create a tube cecostomy if clinically indicated. Dudley also noted that by leaving the tube cecostomy in place, a contrast study could be completed in an antegrade fashion if assessment of the anastomosis is necessary in the postoperative period [27].

More recent studies have described implementation of Dudley et al.'s technique for on-table antegrade colonic lavage, with only slight modification of the catheter location (through the appendix, terminal ileum, or cecum) to deliver irrigation and decrease fecal load proximal to the obstructing lesion in preparation for a primary anastomosis. However, colonic lavage via this technique can be cumbersome and time consuming to perform and is associated with possibility of contamination of spilled fecal content into the operative field [28].

Acknowledging these concerns, Park et al. proposed a one-stage resection and primary anastomosis with intraoperative antegrade irrigation in patients with obstructing left-sided colon cancer. The proximal end of the irrigation device was connected with the dilated colon proximal to the obstructing lesion. The distal end of the device contained two ports: one for drainage of colonic contents and one for insertion of the irrigation catheter. After successful colonic irrigation, an on-table colonoscopy was subsequently performed by inserting a colonoscope through the irrigation port. The incidence of synchronous polyps was 47%. Intraoperative endoscopic detection of a synchronous lesion prompted the surgeon to increase the extent of resection in 17% of patients when biopsy-proven malignancy was determined on frozen section. The device employed in this study was one of the first that facilitated a one-stage procedure in patients with obstructing left-sided colon cancer, as well as enabled a simultaneous on-table colonoscopy to be performed both quickly and easily [28]. 

Determined to decrease incidence of contamination during on-table colonic lavage, Buyukgebiz described a technique utilizing a camera sleeve to control irrigation drainage and spillage from the colon in patients with colonic obstruction due to tumor who underwent one-stage colon resection with primary anastomosis. The colon was divided proximal to the obstructing mass then introduced it into a nylon sleeve traditionally used to sterilely cover intraoperative camera equipment. The sleeve was sutured into place in a telescopic fashion to contain the fecal contents during colonic irrigation and washout via an antegrade catheter inserted through the appendix into the cecum. The in-sleeve method eliminated the need for irrigation drainage catheters, which have diameters smaller than that of the colonic lumen, making them prone to becoming clogged with feculent washout material. Additionally, by washing out the colon, an intraoperative colonoscopy could be effectively performed by introducing the colonoscope through the sleeve and into the transected colon. This technique was found to be a safe and quick means to decrease fecal load in obstructed colons, thus decreasing risk of contamination and allowing proper colon preparation for segmental resection with primary anastomosis. In addition, it provided successful means to perform intraoperative colonoscopy and evaluate the proximal colon on-table for synchronous lesions [19, 29]. 

Intraoperative colonoscopy can be utilized to evaluate the colon that could not be properly endoscopically assessed during the preoperative period. While the entire colon can be manually examined to assess for synchronous lesions at the time of laparotomy, Heald and Bussey reported that up to 69% of synchronous cancer lesions could not be detected by palpation of the colon [30]. Given the high failure rate and lack of sensitivity of operative palpation, intraoperative colonoscopy may serve as a useful adjunct. However, some concerns have been raised regarding on-table colonoscopy; in order to be properly performed, colonoscopy requires distension of the bowel with intraluminal air insufflation that may subsequently distort visualization and compromise the planned procedure, especially during laparoscopic surgery. If the bowel is over distended during insufflation, the colon may be at risk for mucosal, serosal, or mesenteric tears, regional hypoperfusion of tissue, and potential ischemia. Carbon-dioxide may be used for colonoscopic insufflation and offers the advantages of rapid absorption and alleviation of bowel distension when compared to atmospheric, nitrogen-rich air. Nakajima et al. utilized CO_2_-insufflated colonoscopy during laparoscopic colonic resections and found this modality to be feasible, safe, and practical, as it minimized bowel distension without negatively affecting operative exposure or the subsequent procedure. Additionally, it allowed the entire colon to be assessed endoscopically, rather than manually palpated, further facilitating the planned resection to be performed in a minimally invasive fashion [31].

Most recently, Sasaki et al. investigated whether intraoperative colonic irrigation and on-table colonoscopy may be useful for more accurate diagnosis of colorectal cancer before colectomy in patients with completely obstructive left-sided cancer. During a one-stage procedure, intraoperative colonic irrigation was completed using a Y-shaped irrigation catheter consisting of a working port and a drainage tube. After irrigation was completed, an intraoperative colonoscope was inserted through this device, and the bowel proximal to the obstructing tumor was endoscopically examined for presence of synchronous neoplastic lesions. In this study, synchronous adenomatous polyps were detected in 26.8% of patients receiving intraoperative colonic irrigation and colonoscopy, 4.0% of which were determined to be carcinoma on pathologic evaluation. In this study, they reported mean operative time to be 28 minutes longer when on-table colonic irrigation and intraoperative colonoscopy were included in the procedure (271 minutes versus 243 minutes). With intraoperative detection of these synchronous lesions, surgical intervention could be performed during the same procedure, thus deferring the need for a second laparotomy. Sasaki et al. found their protocol to be safe with no mortality, low morbidity, and without significant differences in complication rates [32].

Several studies have also shown that operative planning may need to be altered if a synchronous lesion is present. Arenas et al. conducted a prospective study in an effort to evaluate the need to augment the extent of surgical resection due to detection of a synchronous colorectal lesion. In this series, synchronous lesions were present in 45.6% of patients, with 24% of these lesions influencing the degree of surgical resection. Overall, in 11% of cases, the presence of a synchronous lesion influenced operative management and dictated a more extensive operation than would have been performed with only treating the primary lesion [8]. Kim and Park advocated the use of intraoperative colonoscopy to evaluate for synchronous lesions, reporting that 37.2% of on-table endoscopies performed in their study detected synchronous lesions, leading to additional surgical procedures completed at the time of initial operation in 13.7% of cases in order to remove these lesions [33]. In this notion, the overall goal of operative management should therefore be to perform a procedure with intent to cure, assess for synchronous pathology, and address all pathology present at time of the initial surgical intervention to avoid additional operations.

## 4. Postoperative

While surgical resection is the primary treatment for localized colorectal cancer, development of local, regional, and/or distant recurrence has been reported to occur in 30% to 50% of patients [34]. Greater than 90% of these recurrences have occurred within a 5-year interval following surgery [35]. When detected early, surgical intervention is the treatment of choice for resectable recurrences and for new primary tumors. Recurrence after resection of colorectal cancer is most common in the liver (33%) followed by lung (22%), local recurrence (15% in colon and 35% in rectum), and regional lymph nodes (14%). Less common but still noteworthy is the development of second primary or metachronous lesion in 3% of patients [36]. Though the importance of postoperative surveillance for detection of these lesions is widely acknowledged, no consensus has been established regarding timing and modalities for follow-up protocols in colorectal cancer patients. In 2009, Scheer and Auer performed a literature review exploring current guidelines from various institutions, including the American Society of Clinical Oncology, the National Comprehensive Cancer Network, and the European Society of Medical Oncology, among others. In their meta-analysis, it became apparent that intensive surveillance after curative resection of colorectal cancer improved overall survival, allowing early reoperation to address asymptomatic recurrences [37].

However, different approaches were noted in follow-up protocols, with large variation in frequency of postoperative office visits, serum CEA level monitoring, colonoscopy, and radiologic studies of the chest and abdomen. Office visits are important for surveillance monitoring, allowing the physician to discuss results of investigations, reinforce behavioral modifications, and provide counseling regarding disease process. Advocates of CEA monitoring will trend levels postoperatively, as levels will become elevated in approximately 75% of patients with colorectal recurrence. Depending on the threshold level for abnormal value, sensitivity and specificity for detecting recurrence have been reported between 44% and 80% and 42% and 90%, respectively. Most studies have agreed that CEA monitoring is most sensitive for hepatic and retroperitoneal lesions and least sensitive for local recurrence and pulmonary lesions. Therefore, CEA is used most commonly as a trended value, triggering further work-up if levels are noted to rise [37].

After resection of colorectal cancer, follow-up colonoscopy is recommended to screen recurrence at the anastomosis and to assess metachronous lesions. Mulder et al. reported a significantly higher incidence of metachronous colorectal cancer in patients who had a prior resection for colorectal cancer when compared to an age- and sex-matched population. Of note, these patients had a 1.4 incidence ratio of metachronous lesions within a 3-year interval after initial colorectal cancer diagnosis and resection. The presence of a synchronous lesion at time of initial colorectal cancer resection was noted to be the only significant risk factor for development of metachronous lesion, yielding a relative risk of 13.9 (95% CI 4.7–41.0). Therefore, short-term interval (ideally less than 3 years) surveillance should be recommended for follow-up colonoscopy, possibly sooner for those patients diagnosed with synchronous tumors [38].

Couch and coworkers conducted a retrospective study investigating postoperative colonoscopic surveillance, noting that complete preoperative screening did not translate to a lower incidence of neoplasia detected on first postoperative colonoscopy. Additionally, new neoplastic lesions and recurrences amenable to resection were detected within two years from time of operation. Therefore, Couch et al. advocate a suggested time interval of no more than two years between initial surgery for colorectal cancer and first postoperative surveillance colonoscopy [39]. In their meta-analysis, Scheer and Auer stated that most randomized trials evaluating surveillance for colorectal cancer recurrence after resection of primary lesion reported a median observation period of 5 years or less [37].

Aside from colonoscopy, several imaging modalities are available to assess the colon, as well as extracolonic anatomy. Chest X-ray or thoracic CT can be used if pulmonary lesions are suspected. Abdominal CT and/or ultrasound can be used to image the liver for lesions. American Society of Clinical Oncology current guidelines recommend yearly CT scan of the abdomen for the first 3 years following surgery [37]. A newer imaging modality, CT colonography, may play a role in surveillance after curative resection of colorectal cancer due to its ability to assess for both colonic and extracolonic lesions. Kim and colleagues reported an 81.8% per-patient and 80.8% per-lesion sensitivity for CT colonography when detecting advanced neoplasia, with similar sensitivities of 80% and 78.5%, respectively, for evaluation all adenomatous lesions. CT colonography was noted to have a specificity of 93.1%, with negative predictive values of 100% for adenocarcinoma, 99.1% for advanced neoplasia, and 97% for all adenomatous lesions [40]. More recently, 18FDG-PET has been investigated as a potential diagnostic imaging modality, specifically in patients with an elevated CEA and normal colonoscopy. While this option had a high diagnostic yield for detection of lesions, it is not currently considered a cost-effective method for routine postoperative surveillance [37]. 

As discussed earlier, the need for postoperative colonoscopic surveillance after resection is paramount due to the elevated incidence of synchronous and metachronous lesions in those patients with a history of colorectal malignancy. Typically, in patients who have undergone preoperative colonoscopic evaluation, colonoscopy is performed within one year after colorectal resection. In the patient without a recent preoperative evaluation (within 5 years), who presents with an acute colonic obstruction that necessitates emergency surgical intervention without intraoperative colonic lavage and colonoscopy, it would be recommended to survey that patient in less than one year after resection, preferably 3–6 months postoperatively. In this specific scenario, if a biopsy-proven malignancy or sessile polyp not amenable to endoscopic resection is found on postoperative colonoscopy, the patient will need to be subjected to two abdominal operations for colorectal resection within a 6-month period. Given the age, functional status, and burden of comorbidity of each patient, this predicament can be a challenging one, but one that is potentially avoidable.

## 5. Conclusion

At the current state of technology, multiple modalities exist to visualize the colon in the asymptomatic patient, as well as during operative resection and the perioperative period. Despite this fact, a large portion of the population remains unscreened for colorectal lesions, and high-grade obstruction or other emergent conditions from colonic carcinoma can occur without previous detection. On-table colonic lavage and colonoscopy during the time of resection is a technique that has been utilized for many years but recently is becoming more refined. The goal of recent research is to develop a safe, efficient, high-yield procedure that adds little to overall operating room time and postoperative morbidity while give the surgeon the necessary information to make a proper oncologic decision about the extent of resection to provide our patients the best chances for successful outcome. It is a technique and a technology that requires further use and study but with continued evolution and improvement looks to be a viable option in our intraoperative armamentarium. 

## Figures and Tables

**Table 1:** Alternative strategies for the identification of synchronous colonic lesions for patients that present with acute colonic cancer requiring emergent surgery.

Preoperative	
Virtual CT colonoscopy	
Magnetic resonance colonoscopy	
Colon capsule endoscopy	
Intraoperative	
Subtotal colectomy	
On-table lavage and intraoperative colonoscopy (CO_2_ insufflation)	
